# Cardiovascular Effects of Smoking and Smoking Cessation: A 2024 Update

**DOI:** 10.5334/gh.1399

**Published:** 2025-02-19

**Authors:** Mahfuzur Rahman, Mohammad Alatiqi, Mohammed Al Jarallah, Maryam Yousef Hussain, Abdul Monayem, Prashant Panduranga, Rajesh Rajan

**Affiliations:** 1 Al Amiri Hospital, Kuwait City, Kuwait; 2 Department of Cardiology, Sabah Al Ahmed Cardiac Centre, Al Amiri Hospital, Kuwait City, Kuwait; 3 Royal College of Surgeons in Ireland, Dublin, Ireland; 4 Vitebsk State Medical, University, Vitebsk, Belarus; 5 Department of Cardiology, Royal Hospital, and Director General of Specialized Medical Care, Ministry of Health, Muscat, Oman

**Keywords:** Smoking, Cardiovascular diseases, electronic cigarettes

## Abstract

Smoking is a significant risk factor for both acute and chronic cardiovascular diseases. These diseases contribute to approximately twenty percent of all-cause mortality. Research indicates that quitting smoking can substantially reduce or even reverse the harmful effects associated with smoking on cardiovascular health. Notably, these benefits can be observed in a relatively short period compared to the duration of smoking history. This article aims to provide data to understand the effects of smoking on the cardiovascular system locally as well as its effects as a pandemic globally and hence provide comprehensive strategies in the management of cardiovascular patients for smoking cessation.

## 1. Introduction

Smoking is one of the most common modifiable risk factors for the development of cardiovascular disease (CVD). Despite clear evidence provided since the 1950s, smoking remains a major risk factor for CVD, which is the leading cause of death among adults worldwide (1). According to the World Health Organization (WHO) and Global Heart Journal, smoking is responsible for more than 8 million deaths annually, and one in every five cardiovascular patients dies because of smoking (2,3). Conditions such as coronary artery disease, cerebrovascular disease, aneurysms, and peripheral artery diseases are among the numerous diseases caused by smoking. Both active and passive exposure to smoke inevitably predisposes to cardiac and vascular disorders.

Several factors contribute to the challenge of providing comprehensive data in a single report. Some factors include the amount of exposure to cigarette smoke, the age of onset of smoking, and the subtype of cigarette or tobacco (2,3). This paper aims to provide current status, updates, and evidence of cigarette smoking and its association with CVD for 2024.

## 2. Physiochemical properties of cigarette smoke

Approximately 7,357 chemicals of many different classes can be found either bound to or in free form in the aerosol or gas phase (4). Tar (Total Aerosol Residue) is the weight of solids collected after water and nicotine have been removed. Tar is the sticky brown substance that stains the teeth and turns fingers yellow-brown. The tar is the material that is trapped through the Cambridge glass-fiber filter that retains 99% of all particulate material. The gaseous phase consists of nicotine, which is an addictive substance, but in low doses, it’s relatively harmless, a mild stimulant/relaxant, and carbon monoxide. Chronic carbon monoxide exposure can increase carboxyhemoglobin concentration up to 10% in heavy smokers, producing a functional anemia and related hypoxemia (5). To assess the most significant among these, this paper follows the guidelines of Fowles and Dybing (6), who suggested identifying chemical components with the greatest potential for toxic effects, specifically those associated with cancer, respiratory, and cardiovascular diseases. For CVDs, cyanide, arsenic, and cresols are considered primary risks, while other concerns are N-nitrosamines and polycyclic aromatic hydrocarbons. These concerns, along with Hoffman’s (7) list of biologically active chemicals, can be used to differentiate the toxic chemicals from others in cigarette smoke.

The chemical composition varies between different types of cigarette smoke: mainstream smoke (MS), which is inhaled into the lungs; side-stream smoke (SS), which is released from the burning tip; and second-hand smoke (SHS), which is a mixture of the two. Heavy metals and nitrosamines are present in higher concentrations in SS than in other components. The mean concentration of polycyclic aromatic hydrocarbons (PAHs) is higher in both MS and SS compared to cigarette butts (CBs). The mean phenol concentration is higher in SS compared to MS and CBs (8). The MS also consists of 8% tar and 92% gases. The tar contains >10^17^ free radicals/g, which last hours to months, and the gas contains >10^15^ free radicals/puff, which lasts for a few seconds (9).

Although cigarette smoke is very complex, consisting of over 4,000 compounds linked to CVD, most studies suggest that carbon monoxide, reactive oxygen species, and nicotine are responsible for the pathogenesis of smoking-induced cardiovascular disorders (10,11).

Earlier studies suggested that carbon monoxide (CO) might be correlated with smoking-induced cardiovascular changes, as in hypoxic hypoxia (12). However, more recent studies indicated that CO was an unlikely rationale for the progression of atherosclerosis (13). So far, nicotine is the most studied component. Although nicotine is crucial in increasing cardiac output, pulse rate, and blood pressure, its role in athero-thrombotic diseases is poorly understood (14). Currently, reactive oxygen species (ROS) play a significant role in the development of atherosclerosis. The source of ROS varies between the gas or tar phase of cigarette smoke, monocytes, macrophages, neutrophils, and endogenous sources from xanthine oxidase, endothelial nitric oxide synthase (eNOS), and the mitochondrial electron transport chain (15,16). The detailed role of ROS will be provided in the section on pathogenesis.

### 2.1 Tobacco industry and Tobacco control

Tobacco control is an effective tool that provides comprehensive strategies to protect the people from active as well as SHS. The World Health Organization Framework Convention on Tobacco Control (WHO FCTC), implemented in 2005, is one of the most powerful tools designed to reduce health and economic stress due to tobacco products. The WHO FCTC aims to achieve the sustainable development goals that further visions to reduce the premature mortality due to chronic cardiovascular, respiratory, cancer, and diabetes by one third by 2030 (17). Among the various barriers to successful progression of the tobacco control ideology, the tobacco industry has remained a strong competitive inhibitor to tobacco control’s success.

In the last three decades, the tobacco industry has developed cigarettes that carry lower levels of tar, nicotine, and CO. Since the 1990s, tobacco industries have started advertising products that are to cause less harm to smokers who are health conscious. Although there is very little evidence, some of these products were advertised as nicotine-free (Quest®), lowered carcinogens (Omni®), and reduced hazardous chemicals, specifically in heated, not burned, cigarette-like products (Accord® and Eclipse®). However, in 2006 the U.S. Department of Justice stated the tobacco industry consciously deceived the people through false advertisement claims of light and ultralight cigarettes of having reduced harm compared to flavored or electronic cigarettes. In 2010, tobacco industries were prohibited by the FDA from advertising reduced exposure, such as light and low, on packaging under the Family Smoking Prevention and Tobacco Control Act. However, the tobacco industries still persist in perpetuating misinformation of reduced harm by using colors to depict the lightness of tobacco content in cigarettes (18,19).

Use of these cigarettes or cutting down on the number of cigarettes in reducing harmful effects on health is still controversial. The tar levels in top brand cigarettes range from 1 mg/cig to 13 mg/cig, the nicotine yield from 0.1 mg/cig to 1 mg/cig, and CO levels from 1 mg/cig to 10 mg/cig, which are made mandatory to publish on the cigarette packs (20). In a large study involving 3,500 lung cancer cases, smokers of ultra-low tar cigarettes (machine yield ≤3 mg tar/cigarette) for 8+ years had a somewhat lower risk of lung cancer than those who only smoked high tar cigarettes (≥10 mg tar/cigarette) (21). Thus, the US cancer control department statement opined that some epidemiological studies have found lesser risk of lung cancer among smokers of reduced-yield cigarettes (22). However, they warn that smokers keep cigarette smoke longer in the lungs, increase the speed and depth of inhalation, and increase the number of cigarettes as well when smoking low-tar or low-nicotine cigarettes, in order to increase the nicotine dose. This ‘compensation’ will offset the benefits of low-tar cigarettes.

Reducing consumption of cigarettes per day may reduce harm. It’s found that smoking one instead of 20 cigarettes per day has about one-twentieth (5%) of the risk. This appears to hold true for lung cancer, as demonstrated by the extensive American Cancer Society Prevention Study II, which found a roughly linear relationship between lung cancer risk and cigarette consumption and number of cigarettes smoked per day (23).

Are the above results w.r.t smoking and lung cancer similar to those of cardiovascular disease? There are conflicting results in the literature about low-yield cigarettes benefits concerning cardiovascular diseases. However, in a large meta-analysis of 26 studies, data showed smokers of lower-tar-yield cigarettes having a lower risk than smokers of higher-tar-yield cigarettes. Using estimates adjusted for amount smoked and avoiding overlaps, the risk in the lowest tar group is estimated to be 22% lower for lung cancer and 14% for heart disease than that in the highest tar group (24).

In yet another meta-analysis of 55 studies, smoking only about one cigarette per day carries a risk of developing coronary heart disease and stroke that is about half that for people who smoke 20 per day. In men, the pooled relative risk for coronary heart disease was 1.74 for smoking one cigarette per day and 2.27 for 20 cigarettes per day, and among women, it was 2.1 and 3.95 for one and 20 cigarettes per day, respectively. There was no safe level of smoking for cardiovascular disease (25).

The overall message underscores the significant health risks of smoking, regardless of the type of cigarette or quantity. In the end, even slight smoking raises the likelihood of significant health problems, such as cardiovascular disease and lung cancer. The most effective strategy for maintaining health is to completely refrain from smoking.

## 3. Pathophysiology of Cardiovascular Disorders due to Cigarette smoke

Cigarette smoking is associated with an increased risk of various clinical atherosclerotic syndromes, including acute coronary syndromes, aortic and peripheral arterial disease, cerebrovascular disease, and sudden cardiac death (26). Endothelial dysfunction, inflammation, and a hypercoagulable state are integral to the initiation and progression of atherosclerosis. These factors precede the clinicopathologic manifestation of atherosclerosis (27).

### 3.1 Endothelial dysfunction

**Figure 1:**
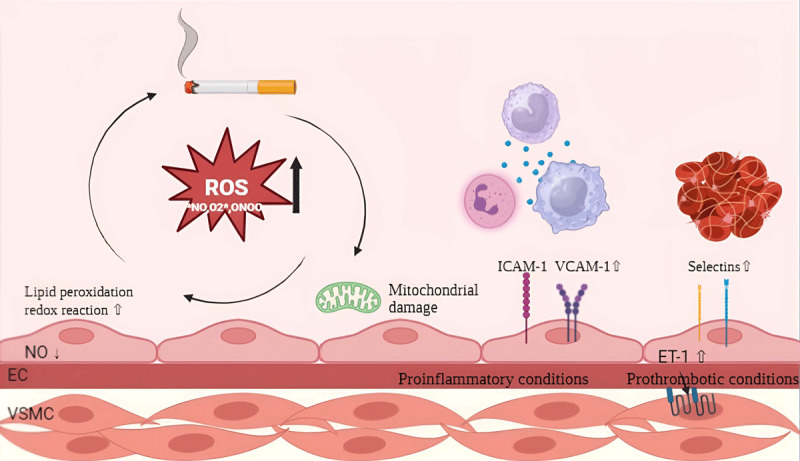
Pathogenesis of endothelial dysfunction. EC, endothelial cells; VSMC, vascular smooth muscle cells; ICAM-1, intercellular adhesion molecule-1; VCAM-1, vascular cell adhesion molecule-1; NO, nitric oxide; ET-1, endothelin 1. Created using Biorender.com.

Various clinical and laboratory studies have shown that endothelial dysfunction induced by cigarette smoke is mediated by decreased nitric oxide (NO) bioavailability, increased production of superoxide anions, and increased production and release of endothelin (28). Reactive oxygen species (ROS) formation and dysfunctional endothelial NO synthase (eNOS) contribute to smoking-induced atherosclerosis. The gas phase of cigarette smoke contains large amounts of free radicals and pro-oxidants such as NO, nitrogen dioxide (NO_2_), phenols, and nitrosamines. In contrast, the tar phase contains large quantities of quinones, which undergo redox cycles to produce superoxide (O_2–_), hydrogen peroxide (H_2_O_2_), and other oxidant species (Figure 1). Superoxide in cigarette smoke is transported through the bloodstream to the vascular endothelium, where it reacts with nitric oxide to form peroxynitrite anion (ONOO–), which is severely cytotoxic (29). Furthermore, the activation of nicotinamide adenine dinucleotide phosphate hydrogen (NADPH) and xanthine oxidase has been shown to increase ROS production in endothelial cells (30,31). Water-soluble tobacco components can cause mitochondrial outer membrane permeabilization (MOMP). Although this does not cause cell death, it does result in the leakage of mitochondrial contents, including mitochondrial DNA and electrolytes, into the cytoplasm. This can lead to ROS production and the release of damage-associated molecular patterns (DAMPs), which are pro-inflammatory molecules (32).

### 3.2 Inflammation

**Figure 2:**
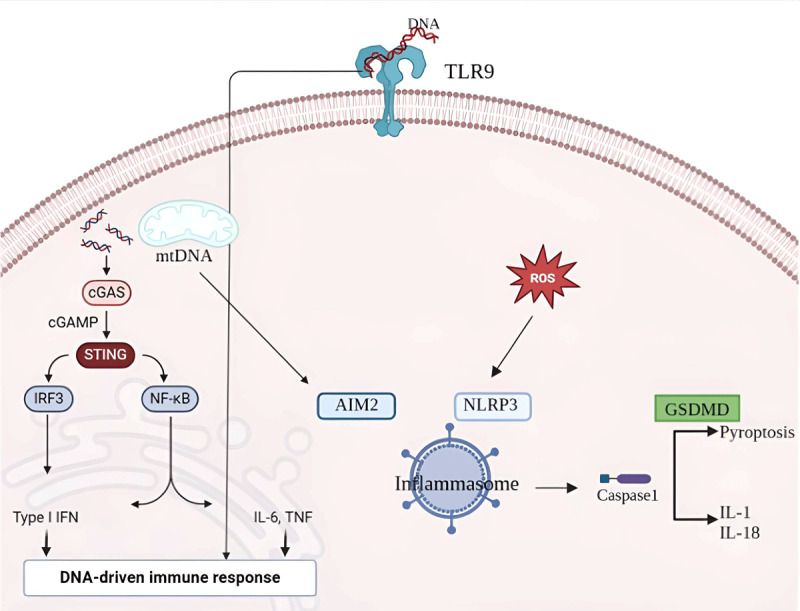
Inflammation at the cellular level induced by smoking. TLR, Toll-like receptor; cGAS, cyclic GMP–AMP synthase; STING, stimulator of interferon genes; mtDNA, mitochondrial DNA; IL, interleukin; ROS, reactive oxygen species; GSDMD, Gasdermin D-mediated pyroptosis. Created using Biorender.com.

Chronic inflammation in the vessel wall is a key factor in the pathogenesis of atherosclerosis, with smoking being the first trigger (Figure 2). At the cytological level, pattern recognition receptors of the innate immune system, particularly Toll-like receptor 9 (TLR9), the NLRP3/AIM2 inflammasome, and cyclic GMP-AMP synthase (cGAS)-stimulator of interferon genes (STING), play crucial roles in the development of vascular lesions.

cGAS is activated by free mitochondrial DNA (mtDNA) released via minor MOMP. Both TLR9 and cGAS-STING pathways lead to increased cytokine synthesis, particularly interleukin-6 (IL-6) and interleukin-8 (IL-8) (33).

Cytosolic mtDNA released via minor MOMP also activates the AIM2 inflammasome, whereas ROS usually activates the NLRP3 inflammasome. These inflammasomes induce gasdermin D (GSDMD)-mediated pyroptosis and subsequent release of cytokines IL-1β and IL-18 (34).

### 3.3 Hypercoagulable State

Cigarette smoke activates endothelial cells, leading to increased expression of ICAM-1, VCAM-1, and selectins (P and E). This promotes the adhesion of monocytes/macrophages, lymphocytes, and platelets to the prothrombotic endothelium. Furthermore, the expression of von Willebrand factor (vWF) on the exposed subendothelium leads to platelet activation, adhesion, and aggregation, resulting in thrombus formation. Chronic smoking also leads to the formation of atheromatous plaques due to increased expression of matrix metalloproteinases (MMP), particularly MMP-12 and MMP-9 in macrophages, MMP-8 and MMP-9 in endothelial cells, and MMP-2 and MMP-9 in vascular smooth muscle cells (35,36).

## 4. Epidemiology

Cardiovascular diseases are the leading category of non-communicable diseases globally, responsible for about one-fifth of all deaths worldwide (2). Modifiable risk factors, such as smoking, contribute to both existing and new cases of CVD. However, the extent of their impact can differ based on the population under study and the methodologies employed in the research (37,38). Tobacco use is also closely linked to an increased risk of early death (39). A more effective reduction in the burden of CVD can be achieved by understanding how regional and gender-specific risk factors contribute to CVD development. The following epidemiological review is based on the Global Cardiovascular Risk Consortium, which has analyzed a harmonized individual-level dataset from population-based cohorts.

The study combined and standardized data from 1,518,028 individuals across 112 cohort studies in 34 countries and 8 geographic regions (North America, Latin America, Western Europe, Eastern Europe and Russia, North Africa, the Middle East, sub-Saharan Africa, Asia, and Australia).

The data was then aligned using variable definitions established by the MONICA/MORGAM project (40) (see Tables 1 and 2). The analysis revealed that 57.2% of new CVD cases in women and 52.6% in men can be attributed to five modifiable risk factors: elevated body mass index, blood pressure, low-density lipoprotein cholesterol, tobacco smoking, and diabetes. The prevalence and influence of these risk factors on new CVD cases differ by gender and geographic location.

**Table 1:** Health study data overview by region (40).


REGION	NUMBER OF PARTICIPANTS	NUMBER OF STUDY COHORTS	RANGE OF SURVEY YEARS	BASELINE AGE (INTERQUARTILE RANGE, YEARS)	PERCENTAGE OF MALES	SMOKING PREVALENCE (%)

**Global**	1,518,028	112	1963–2020	54.4 (45.2 to 63.0)	45.9	21.6

**North America**	65,182	11	1971–2011	54.0 (45.0 to 63.0)	45.9	22.5

**Latin America**	191,244	10	1990–2013	54.0 (45.0 to 63.0)	45.9	30.8

**Western Europe**	907,760	58	1970–2015	54.6 (45.5 to 63.0)	45.9	20.9

**Eastern Europe and Russia**	51,133	16	1983–2014	54.1 (45.5 to 63.0)	45.9	29.2

**North Africa & Middle East**	185,608	5	1963–2020	54.0 (45.0 to 62.6)	45.9	14.2

**Sub-Saharan Africa**	10,390	2	2011–2017	54.0 (45.0 to 63.0)	45.9	18.6

**Asia**	59,802	4	1988–2015	54.0 (45.0 to 63.0)	45.9	23.5

**Australia**	46,909	6	1983–2007	54.6 (45.5 to 63.0)	45.9	14.3


**Table 2:** Population-attributable fraction (PAF) of smoking to cardiovascular diseases (CVD) according to different regions (40).


GEOGRAPHIC REGION	WOMEN (PAF)	MEN (PAF)

	% 10 YEARS OF CVD	% 10 YEARS OF CVD

**Global**	6.7	10.7

**North America**	7.0	7.9

**Latin America**	6.6	9.6

**Western Europe**	8.7	8.6

**Eastern Europe and Russia**	3.0	16.3

**North Africa and Middle East**	4.8	I3.3

**Asia**	5.4	20.2

**Australia**	5.7	5.7


### 4.1 Risk Advancement Periods after smoking cessation

In a recent study, the relationship between the duration of smoking cessation and CVD events was examined by retrospectively analyzing prospectively collected data from the Framingham Heart Study (41). This analysis included participants who were free of CVD at the start and were followed up in time. The findings revealed that heavy smokers (≥20 pack-years) who quit smoking experienced a significantly lower CVD risk within five years compared to current heavy smokers, with a hazard ratio of 0.61, thus confirming the cardiovascular benefits of smoking cessation seen in other research. However, the study also indicated that the reduction in CVD risk occurs very slowly over time. Former heavy smokers only stopped showing a significantly increased CVD risk compared to non-smokers after 10 to 15 years of quitting (5–10 years in the initial cohort and ≥25 years in the offspring cohort). For those with fewer than 20 pack-years of smoking, the excess CVD risk compared to never smokers became insignificant within 10 to 15 years of cessation. This highlights the need to stratify risk advancement periods to avoid underestimating the risk for former heavy smokers after 5 years of cessation.

The risk advancement period refers to the additional time a person must remain smoke-free to counteract the elevated cardiovascular risks caused by prior smoking. An analysis in Heart Journal introduced a method to measure risk advancement periods. Their model calculates the number of years a former smoker needs to remain tobacco-free to achieve cardiovascular risk levels similar to those of individuals who have never smoked. It suggests that each year of smoking adds approximately 2–3 years to the risk advancement period, meaning that former smokers need a longer duration of abstinence than their smoking history to fully mitigate their increased risk (42). Reporting on risk advancement periods involves considering the duration of previous smoking, the time since quitting, and the remaining excess risk compared to non-smokers. A study by Jones et al. provides a framework for reporting these periods. This approach combines traditional risk measures with time-dependent data to estimate how long former smokers need to match the cardiovascular risk levels of never-smokers. The findings indicate that a former smoker who quits at age 40 would generally need about 10–15 years of abstinence to reduce their cardiovascular mortality risk to that of someone who has never smoked (43).

Baseline characteristics of cohort studies standardized by sex and age across different geographic regions. The baseline assessment for all Global Cardiovascular Risk Consortium groups shown here was conducted between 1963 and 2020. 1,518,028 individuals were analyzed using age-and sex-standardized methods.

### 4.2 Females are relatively at higher risk of smoking-induced CVD

Over the past 50 years, the risk of chronic diseases related to smoking in women has significantly increased, with CVD risks now comparable to those seen in men. In a 2011 study, Huxley and Woodward reviewed 8,005 abstracts. They included 26 articles with data on 3,912,809 individuals across 75 cohorts (totalling 2.4 million participants) to estimate the effect of smoking on coronary heart disease in women compared to men, adjusting for other cardiovascular risk factors. They reported a pooled adjusted female-to-male relative risk ratio (RRR) of 1.25 for coronary artery disease (CAD) associated with smoking (44). The specific mechanisms underlying these differences remain unclear, though they may involve factors such as thrombin signaling or variations in smoking behavior between genders. One possible explanation provided by gene expression data in 2024 suggests that smoking is significantly associated with the upregulation of the cytokine receptor-like factor-1 (CRLF1) gene, which translates to actin alpha 2 (ACTA2+) smooth muscle cells, particularly found in carotid plaques. This gene was reported to be upregulated in females compared to males (45).

In a 2013 study by Campesi et al., healthy adult men and women with regular menstrual cycles who were not using oral contraceptives were enrolled. The study found that cigarette smoking led to a more significant reduction in DNA methylation in women compared to men. In addition, women appeared to have increased numbers of platelets, monocytes, lymphocytes, homocysteine, arginine, and asymmetric dimethylarginine (ADMA) levels, which wasn’t observed in men.

Conversely, men showed higher numbers of neutrophils and eosinophils. The study highlighted that smoking has the most severe negative impact on younger females (46).

The Pooling Project on Diet and Coronary Heart Disease found that women aged 40–49 who smoke have a hazard ratio of 8.5 for coronary heart disease compared to non-smokers. In contrast, this ratio decreases to 3.1 for women aged 70 and older. This suggests that cardiovascular risk factors develop earlier in young, healthy female smokers compared to their male counterparts, highlighting the need for more research to explore gender differences in smoking-related health effects. Additionally, women face unique risks associated with smoking, such as those related to pregnancy and the use of oral contraceptives. Therefore, targeted smoking cessation programs for women are urgently needed (47).

## 5. Complications of Smoking on the Cardiovascular System

Complications of smoking can be classified as short-term or long-term.

Under short-term complications, smoking leads to immediate spikes in blood pressure and heart rate. This is primarily due to nicotine’s effect on blood vessels, which causes constriction and temporary cardiovascular stress. A recent study demonstrates that smoking can cause acute elevations in both blood pressure and heart rate (48). These effects contribute to short-term cardiovascular strain and increased risk of acute cardiovascular events. Short-term smoking impairs the endothelial cells lining the blood vessels, disrupting their ability to regulate blood flow and maintain vascular health. Findings show that smoking induces oxidative stress and inflammation that damage the endothelium, compromising vascular function almost immediately (49). Smoking can precipitate an acute myocardial infarction by promoting plaque rupture and increasing blood clotting tendencies. Research featured highlights that smoking is a significant risk factor for acute myocardial infarction, especially in individuals with underlying cardiovascular conditions (50).

Under long-term complications, chronic smoking accelerates the development of atherosclerosis, where fatty deposits build up in the arteries, leading to chronic cardiovascular issues. A comprehensive study reveals that sustained smoking significantly speeds up the progression of atherosclerosis, increasing the risk of severe cardiovascular events over time (51). Prolonged smoking is a major risk factor for developing CAD, characterized by the narrowing of coronary arteries and reduced blood flow to the heart. Evidence indicates that long-term smoking substantially raises the risk of CAD and is linked to poorer outcomes in those already suffering from the disease (52). Chronic smoking contributes to the development of peripheral arterial disease (PAD), a condition where arteries in the legs become narrowed, leading to reduced blood flow and associated complications. A study shows that long-term smoking is closely associated with a higher incidence of PAD, and quitting smoking can significantly improve patient outcomes (53).

In a meta-analysis study comparing multiple prospective cohort studies, tobacco smoking showed a two-fold (past smokers) and a five-fold (current smokers) increased risk of abdominal aortic aneurysm compared to non-smokers. Although the risk reduces with increasing duration of smoking abstinence, the time period can be up to 25 years for the risk to be similar to a non-smoker (54). A study conducted by Zieske et al. in which Patho-biological Determinants of Atherosclerosis in Youth (PDAY) were observed to understand atherosclerosis in trauma victims (15–34 years old). Using the American Heart Association (AHA) classification to describe the lesions of atherosclerosis seen in coronary arteries and the abdominal aorta, smoking individuals showed a significantly higher ratio of advanced lesions (types IV and V) compared to non-smokers (55).

Long-term smoking exacerbates chronic heart failure by contributing to CAD and other cardiovascular disorders. Heart Failure Reviews discusses how smoking accelerates the progression of chronic heart failure and is linked to increased mortality rates among affected individuals (56). Prolonged smoking has also been shown to change the morphology of cardiac muscles, leading to the development of LV hypertrophy from young adulthood to middle age (57). Long-term smoking increases the risk of both ischemic and hemorrhagic strokes by contributing to vascular damage and enhancing clotting tendencies. According to a review, sustained smoking is a significant modifiable risk factor for stroke, with noticeable reductions in risk observed following smoking cessation (58).

## 6. Electronic Cigarettes and vaping products

**Figure 3:**
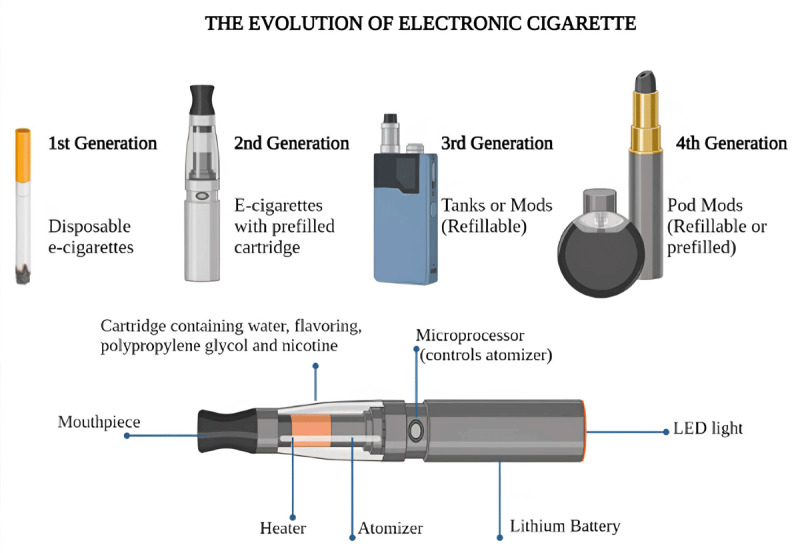
Structure and evolution of electronic cigarettes. E- electronic cigarette (59). Created using Biorender.com.

Electronic cigarette (e-cigarette) and vaping use has seen rapid growth in the past decade, particularly among youth and young adults. The battery-powered electronic device works by heating or vaporizing a liquid solution whose base contains mainly propylene glycol/vegetable glycerin that delivers vaporized nicotine and other flavorings released in aerosols that are inhaled (59) (Figure 3). Other reported compounds, most commonly tetrahydrocannabinol and varying or minute amounts of methamphetamine, methadone, and vitamins, could also be found (60). Currently, all e-cigarettes do not need to undergo premarket animal and human safety studies necessary for drug products, as they are regulated as tobacco products (61).

According to the Nicotine Youth Tobacco Survey (NYTS), electronic nicotine delivery system (ENDS) products are the most commonly used tobacco product among youth in the US. Data from a 2019 analysis by the NYTS showed that around 4.1 million (27.5%) of high school students reported current e-cigarette use compared to 5.8% of combustible cigarettes (62). This data from the US was consistent with similar findings in Canada and England (63). In addition, the US National Health Interview Survey (NHIS) showed a significant increase in ENDS use among adults aged 18–24 from 2014 to 2018. Specifically, current ENDS use among smokers rose from 5.1% to 7.6%, while use among non-smokers increased from 1.5% to 4.6%, and former smokers rose from 10.4% to 36.5% (64).

Current studies on the cardiovascular effects of ENDS are primarily based on short-term research. A systemic review has shown an estimated increase of 2 mm Hg for both systolic and diastolic pressure and 2 bpm for pulse rate in nicotine-containing ENDS product users (65). Although these increases are lower than those observed in combustible smoke users, they are still clinically significant compared to non-users. Cross-sectional studies have shown a higher prevalence of symptoms such as chest pain, palpitations, and arrhythmias in ENDS users (66). Changes to ventricular repolarization are found to be present in combustible smoking as well as ENDS product use (67). Imaging studies via magnetic resonance imaging (MRI) have also shown a reduction in vascular reactivity, blood velocity, and oxidative stress after ENDS use, which suggests its role in endothelial dysfunction and CVD development (68). Although long-term users of ENDS products may show similar endothelial function compared to non-smokers, some studies suggest these endothelial changes as post-use effects (69). A randomized study found increased peripheral blood pressure, heart rate, augmentation index, and pulse wave velocity in users of nicotine-containing ENDS products, similar to effects observed with combustible cigarettes (70). Table 3 shows a comparison of adverse effects among active, passive, and electronic cigarette smoking (71,72,73).

**Table 3:** Comparison of cardiovascular adverse effects in active, passive, and electronic smoking.


	ACTIVE SMOKING	PASSIVE SMOKING	VAPING

**Heart disease**	Significant risk due to plaque buildup and oxidative stress	Increased risk especially with prolonged exposure	Possible increased risk, but lower than smoking

**Stroke**	Significant risk due to hypercoaguable state	Increased risk	Limited evidence

**Hypertension**	Nicotine-induced vasoconstriction	Possible mild increase due to nicotine exposure	Temporary elevation in BP with nicotine-containing e-liquids

**Heart Rate**	Increase in resting pulse due to nicotine stimulation	Possible temporary increase.	Increase in heart rate after vaping with nicotine

**Atherosclerosis**	Accelerated plaque formation	Risk of contributing to plaque formation	Limited data; less risk than smoking

**Oxidative Stress**	Increased oxidative stress contributing to atherosclerosis	Increased oxidative stress from secondhand smoke	Reduced oxidative stress compared to smoking but not eliminated

**Cholesterol**	Increased LDL and decreased HDL	Indirect effects possible with long-term exposure	potential mild effect on lipid profiles


Additionally, platelet dysfunction, leading to a hypercoagulable state, has been observed after exposure to both nicotine-containing and non-nicotine-containing e-liquid aerosols (74). ENDS products also alter coronary flow during stress exercise without affecting myocardial contraction or relaxation (75). Nicotine and ENDS product use can both lower brain glucose utilization and increase vascular stiffness, which may enhance the risk of ischemic brain injury or stroke (76). These effects are more pronounced in women and are thought to be partially due to both nicotine and oral contraceptive use. An in vitro study on female rats demonstrated that nicotine decreases the activity of the aromatase enzyme responsible for estrogen synthesis, which protects the brain from ischemic injury. This suggests that decreased estrogen levels may worsen cerebral ischemia caused by nicotine in women (77).

Clinical studies on the long-term effects of ENDS products are still limited, and more prospective studies are needed. Some cross-sectional case studies have indicated that users, compared to non-users, exhibit increased sympathetic drive, higher low-density lipoprotein (LDL) oxidation, and elevated pro-inflammatory markers, suggesting a higher cardiovascular risk (78).

When discussing management and intervention, limited empirically tested preventive programs for youth ENDS use exist. Such strategies may include the use of novel technology (social media awareness, texting) to advertise ENDS products to youth.

Educational efforts should also extend to parents and healthcare workers, along with other behavioral methods, such as incentives and cognitive behavioral therapy, to effectively promote smoking cessation among youth and young adults (79). Given that ENDS are still relatively new compared to combustible smoking, further development and testing of effective interventions are required to better understand their long-term effects and ensure proper management in the future.

## 7. Management of Smoking Cessation

In 2024, WHO introduced a comprehensive set of tobacco cessation interventions. This includes behavioral support in clinical and community settings through digital tobacco cessation, pharmacological and systemic-level interventions, and policies to maintain the implemented tobacco cessation interventions. For behavioral support, the WHO strongly recommends advice in brief (30 seconds–3 minutes per session), consistently provided as a routine practice by clinicians for all tobacco users. Digital tobacco cessation (text messaging, artificial intelligence-based interventions, or internet-based interventions) was conditionally recommended or used as an adjunct to other cessation interventions or a self-management tool. WHO also made a strong recommendation for all clinicians to include tobacco use status and use of tobacco cessation interventions in their medical records. WHO, as well as the World Heart Federation, strongly recommends increasing and maintaining the adoption of evidence-based treatment interventions (80). In this review, we have briefly discussed the non-pharmacological and pharmacological approaches, as shown below.

### 7.1 Non-pharmacologic management

**Figure 4:**
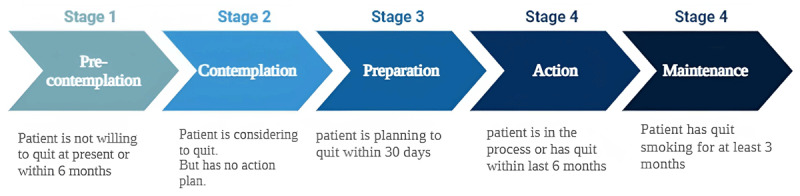
Stages of change in patients undergoing smoking cessation management. Created using biorender.com.

More than 50% of cigarette smokers visit physicians annually in the US alone (81). Identifying them early provides a better chance for clinicians to assess and intervene effectively for smoking cessation. Most patients approaching treatment undergo successive stages of change (Figure 4). Hence, for effective intervention, it is necessary to identify the stage of change under which the patient falls and manage the patient accordingly (82).

**Figure 5:**
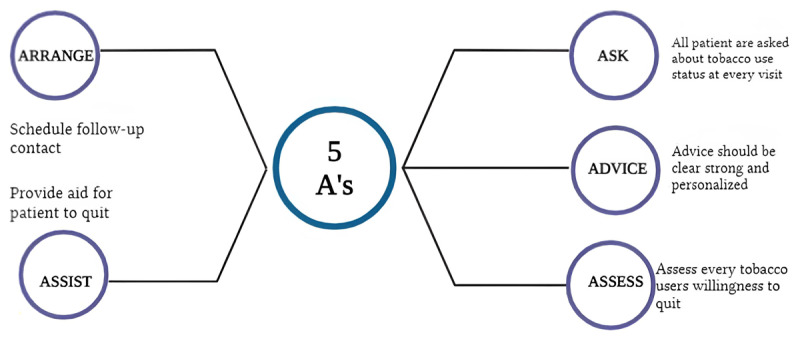
Five A’s algorithm (ask, advise, assess, arrange, assist) in smoking cessation counselling. Created using biorender.com.

**Figure 6:**
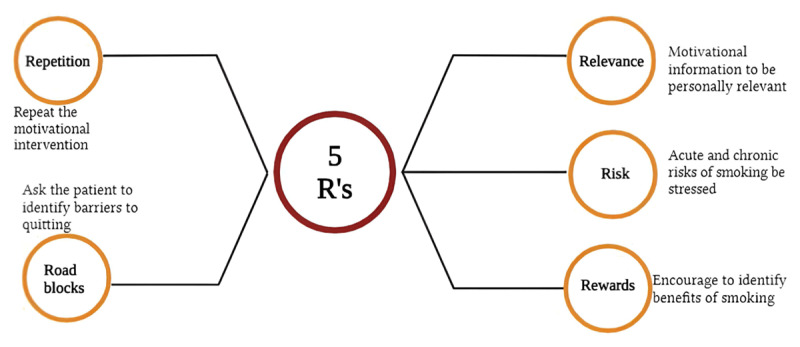
Five R’s algorithm (relevance, risk, rewards, repetition, roadblocks) in motivational interviewing of smoking cessation. Created using biorender.com.

In a primary care setting, physicians can offer a brief counselling intervention using a five-step algorithm called the five A’s (ask, advise, assess, assist, arrange). During the counselling, it might be apparent that patients are unwilling to quit due to a wide range of reasons, from being unaware of cigarette smoking’s harmful effects to not having the financial resources to facilitate smoking cessation (Figure 5). Patients under such circumstances may respond to motivational interventions that are based on the principles of motivational interviewing. To address motivational interviewing, most clinicians can use the five R’s (relevance, risk, rewards, repetition, roadblocks) to summarize the areas for smoking cessation (82) (Figure 6).

Non-pharmacologic interventions for smoking cessation can be mainly categorized under three approaches: clinical, public health, and alternative. Clinical approaches include self-help programs, telephone counselling, cognitive-behavioral therapy, and exercise programs. Public health approaches consist of workplace, multimedia, and community interventions, as well as public policy changes. Alternative approaches include aversive therapy, acupuncture, and hypnosis (44). A systemic review done by Twyman et al. reported the most common barriers to smoking cessation. The review found that lack of support from healthcare providers, smoking for stress management, and acceptability of smoking in vulnerable communities were the three major barriers to smoking cessation among vulnerable groups. It is essential to understand such barriers to smoking cessation and effectively address them by using motivational intervention techniques (83).

Globally, a wide range of tobacco cessation programs are available for patients seeking to quit smoking. Among the various smoking cessation services, in 2004, the Centers for Disease Control and Prevention (CDC), National Cancer Institute (NCI), and Prevention’s Office on Smoking and Health (OSH) formed the National Network of Tobacco Cessation Quitlines (NNTCQ) (84). Quitlines are telephone-based resources that are available in all 50 states of the US. Through the NNTCQ, a person interested in quitting smoking is connected with the state-based resources via NCI’s national access numbers (1–855-DEJELO-YA, 1–800-QUIT-NOW). Other ways to refer a patient would be through the state’s quitline referral system, in which health care providers can send a referral via mail, fax, or electronic health record. Doing so prompts the quitline to directly call the patient, provide feedback, and track patient progress. Quitlines provides its services through counselling, self-help materials, and, if required, referral to other cessation resources. Other than telephone support, text messaging support (National Texting Portal), web-based support (Smokefree.gov), and smartphone apps (quitSTART) are also effective in smoking cessation services. Up to 2024, NNTCQ has successfully provided access to 11.6 million people to cessation services (85). Similar steps were also taken by the National Health Service (NHS) of the UK to implement local stop smoking services. Either via direct (England’s Smoke Free National Helpline on 0300 123 1044) or via referral of GP’s, pharmacists, or other health care providers, an appointment can be made with the smoking cessation service. Services provided include counselling (one-to-one or group), providing smoking cessation aid (pharmacologic therapy), and prevention of relapse through a 4- or 8-week follow-up (86).

### 7.2 Pharmacologic management

All patients willing to quit smoking should be offered pharmacologic treatment unless there are contraindications (82). Currently, seven drugs are approved by the Food and Drug Administration (FDA) for smoking cessation (Table 4): nicotine inhalers, nicotine nasal spray, transdermal nicotine patches, nicotine gum, nicotine lozenges, bupropion sustained-release (SR), and varenicline.

**Table 4:** First line pharmacologic agents for smoking cessation.


GENERIC NAME	DOSE	ADVERSE EFFECTS

**Nicotine Gum**	2 mg gum for <25 cig./day 4 mg gum for >25 cig./day Use up to 12 weeks, no more than 24/day	Jaw pain, mouth soreness, dyspepsia, hiccups

**Nicotine Lozenge**	2 mg lozenge for 1^st^ cig >30 min 4 mg lozenge 1^st^ cig <30 min of waking Use up-to 12 weeks, no more than 20/day	Mouth and throat hiccups

**Nicotine Patch**	>10 cig/day, 21 mg patch for 6–8 weeks <10 cig/day, 14 mg for 6 weeks, 7 mg for 2–4 weeks	Mild skin irritation at placement site

**Nicotine Inhaler**	A dose form consists 1 inhalation. Recommended dosage is 6–16 cartridges/day, up to 6 months	Mouth and throat irritation and cough

**Nicotine Spray**	Spray 1–2 dose/hour Minimum 8 dose/day, maximum 40 dose/day Recommended for 3–6 months	Runny nose, nasal and throat irritation, cough

**Bupropion SR**	Begin 1–2 weeks before quit date. Start at 150 mg for 3 days, then 150 mg twice a day, up to 6 months post-quit	Insomnia, dry mouth, headaches, tremors, nausea, anxiety

**Varenicline**	Start 1 week before quit date at 0.5 mg once daily for 3 days, then 0.5 mg twice daily for 4 days, then 1 mg twice daily for 3–6 months	Nausea, headaches, insomnia, abnormal dreams


These medications are first-line in the management according to US Public Health Service guidelines. Patients who remain refractory or have contraindications to first-line agents may be given second-line agents like clonidine and nortriptyline. Although the FDA has not approved the second-line agents yet, they have shown effectiveness against cigarette smoking (87). Using a second line drug should be considered only after using first-line or if there are contraindications to first-line. A combination therapy of short-acting (nicotine gum, lozenge, inhaler, or spray) with long-acting (nicotine patch or bupropion SR) can be used in the case of patients who remain refractory to monotherapy (87). The treatment must aim to administer an affordable agent that the patient can adhere to with proven efficacy and a good tolerability profile.

#### 7.2a Nicotine Replacement Therapy (NRT)

Among the five NRT products approved by the US FDA for the treatment of tobacco dependence, the nicotine inhaler and spray are prescription drugs in the U.S. In contrast, the other three—nicotine gum, lozenge, and patch—are available over the counter.

NRTs work by partially replacing nicotine obtained by tobacco use, which in turn reduces the severity and duration of withdrawal symptoms (88). A study conducted by a 2008 meta-analysis of 69 clinical trials observed that all five NRT products are superior to placebo with almost double abstinence rates (89). A Cochrane review of 150 trials also found increased quit rates of smoking cessation by 50%–70% after using NRTs (90). Among the adverse effects caused by NRTs, the three most common are reportedly headache, nausea, vomiting, and other gastrointestinal symptoms. Conventionally, NRTs were to be used cautiously in cardiovascular patients, but current studies suggest they are generally safe to use in these patients.

#### 7.2b Bupropion Sustained-Release (SR)

Although bupropion is an inhibitor of dopamine and norepinephrine reuptake, its mechanism in smoking cessation is still not well understood. A systemic review published in 2014 of 44 clinical trials found that monotherapy with bupropion significantly increased smoking abstinence (>6 months) (91). Common adverse effects when used for smoking cessation are insomnia (40%) and dry mouth (10%) (87). A more serious side effect to consider is seizures, as bupropion is said to reduce the seizure threshold. In 2009, the FDA issued alerts to clinicians regarding neuropsychiatric symptoms ranging from agitation to suicidal ideation among patients without preexisting psychiatric symptoms and worsening in psychiatric patients. Hence, the FDA recommends close monitoring of neuropsychiatric symptoms in patients receiving bupropion and to stop bupropion therapy if any contraindications were to be found (92). Following the recommendations of the FDA, a trial conducted by Evins et al. in which they found bupropion, varenicline, and NRTs used among psychiatric patients with psychotic, mood, and anxiety disorders was well tolerated in smoking cessation and superior to placebo (93).

#### 7.2c Varenicline

Varenicline is a partial agonist of the neuronal acetylcholine receptor subtype α4β2 and also functions as an agonist of dopamine turnover, ultimately reducing symptoms of nicotine withdrawal (87). A 2008 meta-analysis showed that varenicline increased quitting rates threefold compared to placebo, with a 33% quit rate at the six-month follow-up (81). In another systematic review of 39 clinical trials, varenicline significantly increased smoking abstinence at six months or longer compared to bupropion (94). Similar findings were observed in the above-mentioned study by Evins et al., where varenicline was superior to both NRTs and bupropion in patients with psychiatric disorders (93). The most common adverse effects reported with its use are nausea, insomnia, and headache. The FDA published a warning in 2011 based on data stating that cardiovascular adverse events were infrequent overall (95,96). Nevertheless, data collected from EVITA trial showed that varenicline was effective in post-ACS patients for smoking cessation (97).

#### 7.2d Clonidine

Clonidine is an α2-adrenergic agonist that is thought to work by counteracting central nervous system (CNS) features of nicotine withdrawal like craving and anxiety. A Cochrane review found that clonidine doubled the rate of abstinence compared to placebo (98). Adverse effects include postural hypotension, drowsiness, fatigue, and dry mouth (87).

#### 7.2e Nortriptyline

Nortriptyline is a tricyclic antidepressant; its mechanism in smoking cessation is not well understood. A meta-analysis review of six randomized clinical trials showed that it doubles the odds of smoking cessation (99). The most common adverse effects are anticholinergic, including dry mouth, constipation, and sedation (87).

#### 7.2f Cytisine

Cytisine, a drug similar to varenicline, is a partial agonist selective for α4β2 nicotinic receptors. A cross-sectional study revealed a smoking abstinence of 50.5% by six months (100). Although it isn’t considered a worldwide pharmacological intervention and is not yet approved by the FDA, it has been used for decades in Western European countries, and it is currently used and reimbursed in some European countries (101,102). However, a randomized clinical trial in the US that is currently in phase 3 has shown promising results in terms of efficacy and tolerability compared to the placebo group (103).

### 7.3 Combination of Pharmacotherapy

According to WHO, there was high-certainty evidence for combination nicotine replacement therapy (CNRT) (a patch plus a short-acting form such as gum or lozenge) in long-term quit rates than NRT alone (104). For varenicline with bupropion/NRT, there was moderate certainty evidence in long-term quit rates compared to varenicline alone; however, due to insufficient evidence for the combination of bupropion with NRT versus NRT alone, WHO has categorized it as low-certainty evidence (105).

In a study conducted in a double-blind, placebo-controlled, sequential, multiple assignment randomized trial (SMART), patients who didn’t respond initially to monotherapy like varenicline or combination therapy with CNRT were later shown to achieve abstinence via increasing the dosage of varenicline or patients who were already on CNRT to either increase the dose or to switch with varenicline (106).

### 7.4 Treatment recommendations for cardiovascular patients

Patients with CVD should abstain from smoking during the acute phase of their condition. Additionally, it is important to note the contraindications of NRT during the first 48 hours post-event of CVD (107).

**Figure 7:**
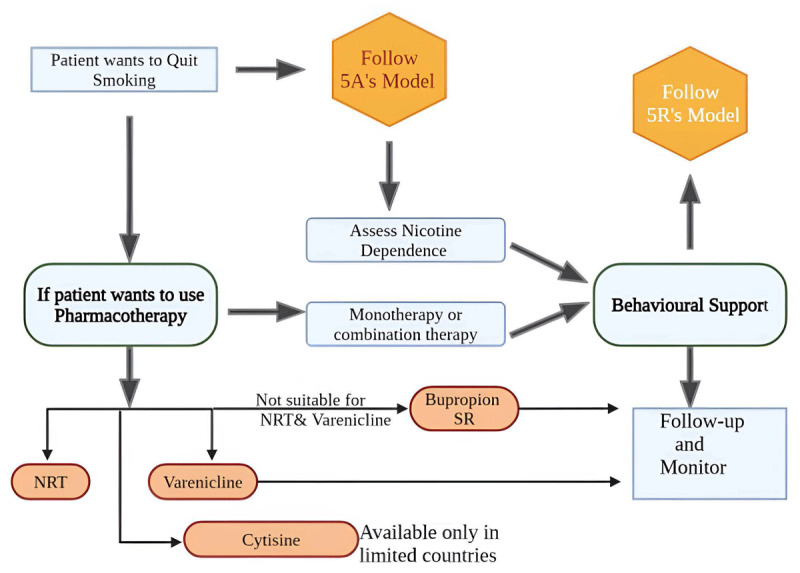
Guidelines for smoking cessation according to the European Network for Smoking and Tobacco Prevention (ENSP) (102), bupropion SR, NRT. Created using Biorender.com.

**Figure 8:**
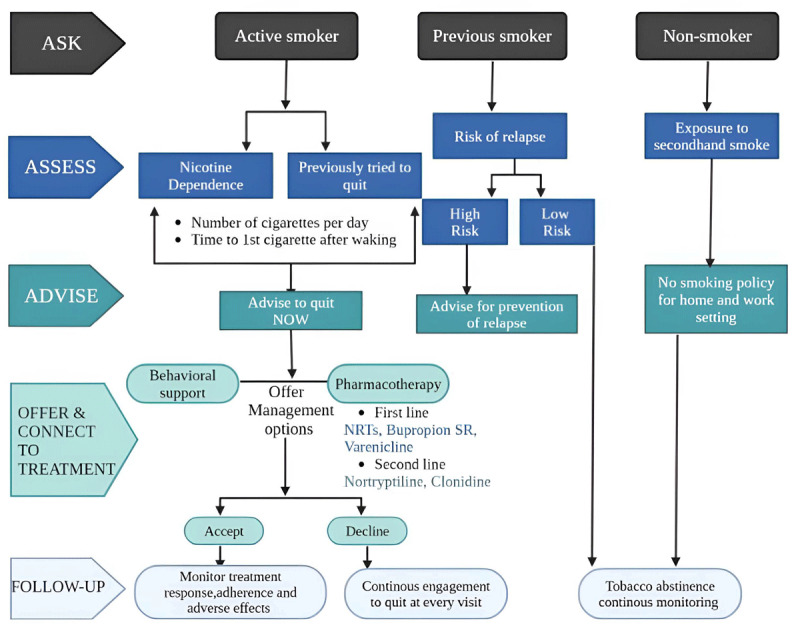
Guidelines for smoking cessation according to the Journal of American College of Cardiology (JACC) (110) bupropion SR, NRT. Created using Biorender.com.

Pharmacotherapy becomes more effective when coupled with behavioral interventions. One of the drugs to consider in this case is varenicline, which is shown to be reasonably safe in cases of chronic, stable CAD with no psychiatric disorders (108). In addition, a biopsychosocial approach is recommended rather than medication alone (102). Also reported in many studies is that the implementation of laws to maintain clean indoor air in hospitals has shown a significant decline in the admission of cardiac disease patients in hospitals (109). Conclusively, in management, we reviewed two international guidelines for smoking cessation by the European Network for Smoking and Tobacco Prevention (ENSP) and the Journal of American College of Cardiology (JACC) (110) (Figures 7, 8).

## 8. Post-smoking cessation

Quitting smoking yields significant improvements in cardiovascular health almost immediately. In the initial weeks after cessation, blood pressure and heart rate stabilize, and the risk of acute cardiovascular incidents declines markedly. A study published highlights that individuals who stop smoking experience a notable decrease in the occurrence of acute myocardial infarction and stroke within the first year. The research also demonstrates that cardiovascular risk declines substantially within months after quitting (111). Long-term cessation significantly lowers the risk of chronic cardiovascular diseases, such as CAD and heart failure.

Over time, the risk of cardiovascular-related mortality approaches that of individuals who have never smoked. According to a longitudinal study, the risk of CVD and mortality continues to decrease progressively with sustained smoking cessation. After five years without smoking, the risk of CAD and stroke is considerably lower compared to those who continue smoking. By 10 to 15 years of abstinence, the risk of cardiovascular mortality becomes comparable to that of never-smokers (112).

Smoking cessation is commonly linked with weight gain, with individuals typically experiencing an increase of about 4–5 kg within the first year after quitting. Additionally, there may be a decline in glucose and lipid metabolism, and weight gain can often lead individuals to resume smoking. One study investigated the factors related to weight gain following smoking cessation by analyzing data collected during the initial visit to a smoking cessation clinic (113). No significant link was found between smoking-cessation-induced weight gain and worsening prognosis of CVDs.

Among various biomolecular studies, the alpha1-antitrypsin LDL (AT-LDL) complex, which consists of alpha1-antitrypsin (AT) bound to oxidized LDL, is a significant cardiovascular biomarker. Smokers typically have much higher serum levels of AT-LDL compared to non-smokers. However, studies have shown that AT-LDL levels decrease significantly within three months after quitting smoking. In essence, weight gain within the first three months of quitting may hinder improvements in AT-LDL levels. Nevertheless, one year after quitting, serum AT-LDL levels decreased more significantly than at the three-month mark, regardless of any increase in obesity (114,115,116).

Research has found that individuals without diabetes experience a significant reduction in CVD risk within four years of quitting smoking, even if they gain a considerable amount of weight. Conversely, individuals with diabetes saw a decreased risk of CVD if they remained smoke-free for at least four years and gained no more than 5 kg (117). Hence, such findings emphasize that weight gain, particularly for secondary risk factors of CVD such as diabetes, might require additional management relative to the less morbid risk groups.

In summary, weight gain often occurs after quitting smoking. While obesity by itself is a significant risk factor of CVDs, the transient weight change due to smoking cessation does not hinder the advantages of quitting smoking that will eventually provide greater long-term benefits (118).

## 9. Conclusions

Cardiovascular diseases are currently the leading cause of non-communicable diseases globally, with modifiable factors such as smoking contributing to both existing and new cases. Both combustible and electronic cigarettes can affect health in numerous ways. For effective global management and reduction, it is essential to study regional distribution as well as age and gender factors; they are the foundational level for evidence-based interventions, which are strongly recommended by WHO and the World Heart Federation. The complications of smoking on the cardiovascular system can range from acute life-threatening emergencies to chronic irreversible health issues. Effective management incorporates both pharmacological and behavioral therapies to treat smoking dependence. From literature review, although cardiovascular risk is reduced in smokers of low-tar or low-nicotine cigarettes or by reducing the number of cigarettes, the estimated reductions in risk are much less than the increase in risk associated with cigarette smoking when compared to never-smoked individuals. Although post-cessation challenges, such as weight gain, may arise, the overall benefits significantly outweigh the major risks and mortality associated with smoking. This paper aims to enhance our understanding of smoking and improve treatment strategies for cardiovascular patients who smoke.

## Abbreviations

1. ACTA2+ – actin alpha 2
2. ADMA – asymmetric dimethylarginine
3. AHA – American Heart Association
4. AT – alpha1-antitrypsin
5. AT-LDL – alpha1-antitrypsin low-density lipoprotein
6. Bupropion SR – bupropion sustained-release
7. CAD – coronary artery disease
8. CBs – cigarette butts
9. CDC – Centers for Disease Control and Prevention
10. CNRT – combined nicotine replacement therapy
11. CNS – central nervous system
12. CO – carbon monoxide
13. CRLF1 – cytokine receptor-like factor-1
14. CVD – cardiovascular disease
15. DAMPs – damage-associated molecular patterns
16. EC – endothelial cells
17. E-cigarette – electronic cigarette
18. ENDS – electronic nicotine delivery system
19. eNOS – endothelial nitric oxide synthase
20. ENSP – European Network for Smoking and Tobacco Prevention
21. ET-1 – endothelin 1
22. FCTC – Framework Convention on Tobacco Control
23. FDA – Food and Drug Administration
24. GSDMD – Gasdermin D-mediated pyroptosis
25. H_2_O_2_ – hydrogen peroxide
26. ICAM-1 – intercellular adhesion molecule-1
27. IL – interleukin
28. JACC – Journal of American College of Cardiology
29. LDL – low density lipoprotein
30. MOMP – mitochondrial outer membrane permeabilization
31. MRI – magnetic resonance imaging
32. MS – mainstream smoke
33. mtDNA – mitochondrial deoxyribonucleic acid
34. NADPH – nicotinamide adenine dinucleotide phosphate hydrogen
35. NCI – National Cancer Institute
36. NHIS – National Health Interview Survey
37. NHS – National Health Service
38. NNTCQ – National Network of Tobacco Cessation Quitlines
39. NO – nitric oxide
40. NO_2_ – nitrogen dioxide
41. NRT – nicotine replacement therapy
42. NYTS – Nicotine Youth Tobacco Survey
43. O_2–_ – superoxide
44. ONOO – peroxynitrite anion
45. OSH – Office on Smoking and Health
46. PAD – peripheral arterial disease
47. PAF – population-attributable fraction
48. PAHs – polycyclic aromatic hydrocarbons
49. ROS – reactive oxygen species
50. SHS – second-hand smoke
51. SMART – sequential multiple assignment randomized trial
52. SS – side-stream smoke
53. STING – stimulator of interferon genes
54. TAR – total aerosol residue
55. TLR – toll-like receptor
56. VCAM-1 – vascular cell adhesion molecule-1
57. VSMC – vascular smooth muscle cells
58. VWF – von Willebrand factor
59. WHO – World Health Organization

## References

[1] Benjamin EJ, Virani SS, Callaway CW, Chamberlain AM, Chang AR, Cheng S, et al. Heart disease and stroke statistics—2018 update: A report from the American Heart Association. Circulation. 2018; 137(12):e67–e492. DOI: 10.1161/CIR.0000000000000558 29386200

[2] World Health Organization. WHO clinical treatment guideline for tobacco cessation in adults. World Health Organization; Jul 2024. 53 p. Available from: https://www.who.int/publications/i/item/9789240096431. 38954657

[3] Dalmau R, Alanazi AM, Arora M, Banerjee A, Bianco E, Gaalema DE, et al. A complex interplay: Navigating the crossroads of tobacco use, cardiovascular disease, and the COVID-19 pandemic: A WHF policy brief. Global Heart. 2024; 19(1):55. DOI: 10.5334/gh.1334 38973985 PMC11225561

[4] Rodgman A, Perfetti TA. The chemical components of tobacco and tobacco smoke. Boca Raton, FL: CRC Press, Taylor & Francis Group; 2009. DOI: 10.1201/9781420078848

[5] Pryor WA, Stone K. Oxidants in cigarette smoke: Radicals, hydrogen peroxide, peroxynitrate, and peroxynitrite. Annals of New York Academy of Sciences. 1993; 686(1993):12–28. DOI: 10.1111/j.1749-6632.1993.tb39148.x 8512242

[6] Fowles J, Dybing E. Application of toxicological risk assessment principles to the chemical constituents of cigarette smoke. Tobacco Control. 2003; 12(4):424–430. DOI: 10.1136/tc.12.4.424 14660781 PMC1747794

[7] Hoffmann D, Hoffmann I. Letters to the Editor – Tobacco smoke components. Beiträge zur Tabakforschung International. 1998; 18(1):49–52. DOI: 10.2478/cttr-2013-0668

[8] Soleimani F, Dobaradaran S, De-la-Torre GE, Schmidt TC, Saeedi R. Content of toxic components of cigarette, cigarette smoke vs Cigarette Butts: A comprehensive systematic review. Science of the Total Environment. 2022; 813(2022):152667. DOI: 10.1016/j.scitotenv.2021.152667 34963586

[9] Ambrose JA, Barua RS. The pathophysiology of cigarette smoking and cardiovascular disease: an update. Journal of the American College of Cardiology. 2004; 43(10):1731–1737. DOI: 10.1016/j.jacc.2003.12.047 15145091

[10] Smith CJ, Fischer TH. Particulate and vapor phase constituents of cigarette mainstream smoke and risk of myocardial infarction. Atherosclerosis. 2001; 158(2):257–267. DOI: 10.1016/s0021-9150(01)00570-6 11583703

[11] Barua RS, Ambrose JA. Mechanisms of coronary thrombosis in cigarette smoke exposure. Arteriosclerosis, Thrombosis, and Vascular Biology. 2013; 33(7):1460–1467. DOI: 10.1161/ATVBAHA.112.300154 23685556

[12] Kjeldsen K, Thomsen HK, Astrup P. Effects of carbon monoxide on myocardium: Ultrastructural changes in rabbits after moderate, chronic exposure. Circulation Research. 1974; 34(3):339–348. DOI: 10.1161/01.res.34.3.339 4818758

[13] Zevin S, Saunders S, Gourlay SG, Jacob P, Benowitz NL. Cardiovascular effects of carbon monoxide and cigarette smoking. Journal of the American College of Cardiology. 2001; 38(6):1633–1638. DOI: 10.1016/s0735-1097(01)01616-3 11704374

[14] Sun YP, Zhu BQ, Browne AE, Sievers RE, Bekker JM, Chatterjee K, et al. Nicotine does not influence arterial lipid deposits in rabbits exposed to second-hand smoke. Circulation. 2001; 104(7):810–814. DOI: 10.1161/hc3301.092788 11502707

[15] Svrivastava SK, Barua RS, Saha DC, Eales-Reynolds LJ, DeVoe MC, Ambrose JA. Endogenous free radical generating sources are involved in smoking-mediated dysfunction of nitric oxide biosynthesis in human coronary artery endothelial cells: An in vitro demonstration, Journal of the American College of Cardiology. 2003; 41(6 Supplement 1):306A. DOI: 10.1016/S0735-1097(03)82432-4

[16] Kayyali US, Budhiraja R, Pennella CM, Cooray S, Lanzillo JJ, Chalkley R, et al. Upregulation of xanthine oxidase by tobacco smoke condensate in pulmonary endothelial cells. Toxicology and Applied Pharmacology. 2003; 188(1):59–68. DOI: 10.1016/s0041-008x(02)00076-5 12668123

[17] Meier BM, Shelley D. The fourth pillar of the Framework Convention on Tobacco Control: harm reduction and the international human right to health. Public Health Reports. 2006; 121(5):494–500. DOI: 10.1177/003335490612100503 16972501 PMC1564445

[18] Hatsukami DK, Carroll DM. Tobacco harm reduction: Past history, current controversies and a proposed approach for the future. Preventive Medicine. 2020; 140:106099. DOI: 10.1016/j.ypmed.2020.106099 32335031 PMC7581601

[19] Congress US. Family Smoking Prevention and Tobacco Control Act. In Congress US (Ed.) H R 1256 Vol Public Law. Washington, DC: U. S. Government Printing Office; 2009. pp. 111–131.

[20] European Union. Manufacture, presentation and sale of tobacco products [Internet]. EUR-Lex [updated 2014 Jun; cited 2024 Sept]. Available from: https://eur-lex.europa.eu/EN/legal-content/summary/manufacture-presentation-and-sale-of-tobacco-products.html.

[21] Lee PN, Fry JS, Hamling JS. Investigation into the risk of ultra-low tar cigarettes and lung cancer. Regulatory Toxicology and Pharmacology. 2017; 89(2017):112–117. DOI: 10.1016/j.yrtph.2017.07.023 28751259

[22] National Cancer Institute. Risks associated with smoking cigarettes with low machine-measured yields of tar and nicotine. Bethesda, MD: US Department of Health and Human Services, National Institutes of Health, National Cancer Institute; 2001 Oct. Available from: https://cancercontrol.cancer.gov/sites/default/files/2020-08/m13_complete.pdf.

[23] Pope CA 3rd, Burnett RT, Turner MC, Cohen A, Krewski D, Jerrett M, et al. Lung cancer and cardiovascular disease mortality associated with ambient air pollution and cigarette smoke: Shape of the exposure-response relationships. Environmental Health Perspectives. 2011; 119(11):1616–1621. DOI: 10.1289/ehp.1103639 21768054 PMC3226505

[24] Lee PN. Tar level of cigarettes smoked and risk of smoking-related diseases. Inhalation Toxicology. 2018; 30(1):5–18. DOI: 10.1080/08958378.2018.1443174 29488428

[25] Hackshaw A, Morris JK, Boniface S, Tang JL, Milenković D. Low cigarette consumption and risk of coronary heart disease and stroke: Meta-analysis of 141 cohort studies in 55 study reports. BMJ. 2018; 360:j5855. DOI: 10.113.6/bmj.j5855 29367388 PMC5781309

[26] Black HR. Smoking and cardiovascular disease. In Laragh JH, Brenner BM (Eds.) Hypertension: Pathophysiology, Diagnosis and Management. 2nd edition. New York, NY: Raven Press Ltd.; 1995. pp. 2621–2647.

[27] Clarkson TB, Weingand KW, Kaplan JR, Adams MR. Mechanisms of atherogenesis. Circulation. 1987; 76(1 part 2):20–28. 3297405

[28] Rahman MM, Laher I. Structural and functional alteration of blood vessels caused by cigarette smoking: An overview of molecular mechanisms. Current Vascular Pharmacology. 2007; 5(4):276–292. DOI: 10.2174/157016107782023406 17979794

[29] Barua RS, Ambrose JA, Srivastava S, DeVoe MC, Eales-Reynolds LJ. Reactive oxygen species are involved in smoking-induced dysfunction of nitric oxide biosynthesis and upregulation of endothelial nitric oxide synthase: An in vitro demonstration in human coronary artery endothelial cells. Circulation. 2003; 107(18):2342–2347. DOI: 10.1161/01.CIR.0000066691.52789.BE 12707237

[30] Kayyali US, Budhiraja R, Pennella CM, Cooray S, Lanzillo JJ, Chalkley R, et al. Upregulation of xanthine oxidase by tobacco smoke condensate in pulmonary endothelial cells. Toxicology and Applied Pharmacology. 2003; 188(1):59–68. DOI: 10.1016/s0041-008X(02)00076-5 12668123

[31] Jaimes EA, DeMaster EG, Tian RX, Raij L. Stable compounds of cigarette smoke induce endothelial superoxide anion production via NADPH oxidase activation. Arteriosclerosis, Thrombosis, and Vascular Biology. 2004; 24(6):1031–1036. DOI: 10.1161/01.ATV.0000127083.88549.58 15059808

[32] Suski JM, Lebiedzinska M, Bonora M, Pinton P, Duszynski J, Wieckowski MR. Relation between mitochondrial membrane potential and ROS formation. In Palmeira CM, Moreno AJ (Eds.) Mitochondrial Bioenergetics: Methods and Protocols. Totowa, NJ: Humana Press; 2012. pp. 183–205. DOI: 10.1007/978-1-61779-382-0_12 22057568

[33] Hansson GK, Libby P, Schönbeck U, Yan ZQ. Innate and adaptive immunity in the pathogenesis of atherosclerosis. Circulation Research. 2002; 91(4):281–291. DOI: 10.1161/01.res.0000029784.15893.10 12193460

[34] Ishida M, Sakai C, Ishida T. Role of DNA damage in the pathogenesis of atherosclerosis. Journal of Cardiology. 2022; 81(4):331–336. DOI: 10.1016/j.jjcc.2022.08.010 36109257

[35] Bouki KP, Katsafados MG, Chatzopoulos DN, Psychari SN, Toutouzas KP, Charalampopoulos AF, et al. Inflammatory markers and plaque morphology: An optical coherence tomography study. International Journal of Cardiology. 2012; 154(3):287–292. DOI: 10.1016/j.ijcard.2010.09.059 20974497

[36] Hawkins RI. Smoking, platelets and thrombosis. Nature. 1972; 236(5348):450–452. DOI: 10.1038/236450a0 4555360

[37] Global Cardiovascular Risk Consortium; Magnussen C, Ojeda FM, Leong DP, Alegre-Diaz J, Amouyel P, Aviles-Santa L, et al. Global effect of modifiable risk factors on cardiovascular disease and mortality. The New England Journal of Medicine. 2023; 389(14):1273–1285. DOI: 10.1056/NEJMoa2206916 37632466 PMC10589462

[38] Yusuf S, Joseph P, Rangarajan S, Islam S, Mente A, Hystad P, et al. Modifiable risk factors, cardiovascular disease, and mortality in 155 722 individuals from 21 high-income, middle-income, and low-income countries (PURE): A prospective cohort study. Lancet. 2020; 395(10226):795–808. DOI: 10.1016/S0140-6736(19)32008-2 31492503 PMC8006904

[39] Jaspers NEM, Blaha MJ, Matsushita K, van der Schouw Y, Wareham NJ, Khaw KT, et al. Prediction of individualized lifetime benefit from cholesterol lowering, blood pressure lowering, antithrombotic therapy, and smoking cessation in apparently healthy people. European Heart Journal. 2020; 41(11):1190–1199. DOI: 10.1093/eurheartj/ehz239 31102402 PMC7229871

[40] Evans A, Salomaa V, Kulathinal S, Asplund K, Cambien F, Ferrario M, et al. MORGAM (an international pooling of cardiovascular cohorts). International Journal of Epidemiology. 2005; 34(1):21–27. DOI: 10.1093/ije/dyh327 15561751

[41] Duncan MS, Freiberg MS, Greevy, RA Jr., Kundu S, Vasan RS, Tindle HA. Association of smoking cessation with subsequent risk of cardiovascular disease. JAMA. 2019; 322(7):642–650. DOI: 10.1001/jama.2019.10298 31429895 PMC6704757

[42] Walicka M, Krysiński A, La Rosa GRM, Sun A, Campagna D, Di Ciaula A, et al. Influence of quitting smoking on diabetes-related complications: A scoping review with a systematic search strategy. Diabetes & Metabolic Syndrome. 2024; 18(5):103044. DOI: 10.1016/j.dsx.2024.103044 38810420

[43] Jones MR, Barnoya J, Stranges S, Losonczy L, Navas-Acien A. Cardiovascular events following smoke-free legislations: An updated systematic review and meta-analysis. Current Environmental Health Reports. 2014; 1(3):239–249. DOI: 10.1007/s40572-014-0020-1 25328861 PMC4198310

[44] Huxley RR, Woodward M. Cigarette smoking as a risk factor for coronary heart disease in women compared with men: a systematic review and meta-analysis of prospective cohort studies. Lancet. 2011; 378(9799):1297–1305. DOI: ttps://doi.org/10.1016/S0140-6736(11)60781-2 21839503 10.1016/S0140-6736(11)60781-2

[45] Lan T, Palm KCA, Hoeben L, Benavente ED, Perry RN, Civelek M, et al. Tobacco smoking is associated with sex- and plaque-type specific upregulation of CRLF1 in atherosclerotic lesions. Atherosclerosis. 2024; 397(2024):118554. DOI: 10.1016/j.atherosclerosis.2024.118554 39137621

[46] Campesi I, Carru C, Zinellu A, Occhioni S, Sanna M, Palermo M, et al. Regular cigarette smoking influences the transsulfuration pathway, endothelial function, and inflammation biomarkers in a sex-gender specific manner in healthy young humans. American Journal of Translational Research. 2013; 5(5):497–509. 23977409 PMC3745437

[47] Tolstrup JS, Hvidtfeldt UA, Flachs EM, Spiegelman D, Heitmann BL, Bälter K, et al. Smoking and risk of coronary heart disease in younger, middle-aged, and older adults. American Journal of Public Health. 2014; 104(1):96–102. DOI: 10.2105/AJPH.2012.301091 23763425 PMC3910023

[48] Dimitriadis K, Narkiewicz K, Leontsinis I, Konstantinidis D, Mihas C, Andrikou I, et al. Acute effects of electronic and tobacco cigarette smoking on sympathetic nerve activity and blood pressure in humans. International Journal of Environmental Research and Public Health. 2022; 19(6):3237. DOI: 10.3390/ijerph19063237 35328926 PMC8952787

[49] Münzel T, Hahad O, Kuntic M, Keaney JF, Deanfield JE, Daiber A. Effects of tobacco cigarettes, e-cigarettes, and waterpipe smoking on endothelial function and clinical outcomes. European Heart Journal. 2020; 41(41):4057–4070. DOI: 10.1093/eurheartj/ehaa460 32585699 PMC7454514

[50] Barkhurdar W, Hussain A, Saqlain M, Zahra R, Hussain I, Saqib M, et al. Smoking behaviour in post-acute myocardial infarction patients: cross-sectional study. Annals of Medicine and Surgery. 2024; 86(5):2531–2537. DOI: 10.1097/MS9.0000000000001333 38694391 PMC11060240

[51] Wu X, Zhang H, Qi W, Zhang Y, Li J, Li Z, et al. Nicotine promotes atherosclerosis via ROS-NLRP3-mediated endothelial cell pyroptosis. Cell Death & Disease. 2018; 9(2):171. DOI: 10.1038/s41419-017-0257-3 29416034 PMC5833729

[52] Salehi N, Janjani P, Tadbiri H, Rozbahani M, Jalilian M. Effect of cigarette smoking on coronary arteries and pattern and severity of coronary artery disease: A review. The Journal of International Medical Research. 2021; 49(12):3000605211059893. DOI: 10.1177/03000605211059893 34855538 PMC8647272

[53] Behrooz L, Abumoawad A, Rizvi SHM, Hamburg NM. A modern day perspective on smoking in peripheral artery disease. Frontiers in Cardiovascular Medicine. 2023; 10:1154708. DOI: 10.3389/fcvm.2023.1154708 37187787 PMC10175606

[54] Aune D, Schlesinger S, Norat T, Riboli E. Tobacco smoking and the risk of abdominal aortic aneurysm: A systematic review and meta-analysis of prospective studies. Scientific Reports. 2018; 8(1):14786. DOI: 10.1038/s41598-018-32100-2 30283044 PMC6170425

[55] Zieske AW, Takei H, Fallon KB, Strong JP. Smoking and atherosclerosis in youth. Atherosclerosis. 1999; 144(2):403–408. DOI: 10.1016/s0021-9150(98)00326-8 10407501

[56] Virani SS, Newby LK, Arnold SV, Bittner V, Brewer LC, Demeter SH, et al. 2023 AHA/ACC/ACCP/ASPC/NLA/PCNA guideline for the management of patients with chronic coronary disease: A report of the American Heart Association/American College of Cardiology Joint Committee on Clinical Practice Guidelines. Circulation. 2023; 148(9):e9–e119. DOI: 10.1161/CIR.0000000000001168 37471501

[57] Gidding SS, Liu K, Colangelo LA, Cook NL, Goff DC, et al. Longitudinal determinants of left ventricular mass and geometry: the Coronary Artery Risk Development in Young Adults (CARDIA) study. Circulation Cardiovascular Imaging. 2013; 6(5):769–775. DOI: 10.1161/CIRCIMAGING.112.000450 23922005 PMC3873157

[58] N’Gbo N’Gbo Ikazabo R, Bier JC, Jamart J, Mostosi C, Mavroudakis N. Impact on quitting smoking of cognitive impairment in stroke patients. Journal of the Neurological Sciences. 2022; 439(2022):120296. DOI: 10.1016/j.jns.2022.120296 35640330

[59] Benowitz NL, Fraiman JB. Cardiovascular effects of electronic cigarettes. Nature Reviews Cardiology. 2017; 14(8):447–456. DOI: 10.1038/nrcardio.2017.36 28332500 PMC5519136

[60] Mulder HA, Patterson JL, Halquist MS, Kosmider L, Turner JBM, Poklis JL, et al. The effect of electronic cigarette user modifications and e-liquid adulteration on the particle size profile of an aerosolized product. Scientific Reports. 2019; 9(1):10221. DOI: 10.1038/s41598-019-46387-2 31308389 PMC6629610

[61] Gottlieb MA. Regulation of e-cigarettes in the United States and its role in a youth epidemic. Children. 2019; 6(3):40. DOI: 10.3390/children6030040d 30836677 PMC6463025

[62] Cullen KA, Gentzke AS, Sawdey MD, Chang JT, Anic GM, Wang TW, et al. E-cigarette use among youth in the United States, 2019. JAMA. 2019; 322(21):2095–2103. DOI: 10.1001/jama.2019.18387 31688912 PMC6865299

[63] Hammond D, Reid JL, Rynard VL, Fong GT, Cummings KM, McNeill A, et al. Prevalence of vaping and smoking among adolescents in Canada, England, and the United States: Repeat national cross sectional surveys. BMJ. 2019; 365:l2219. DOI: 10.1136/bmj.l2219 31221636 PMC6582265

[64] Dai H, Leventhal AM. Prevalence of e-cigarette use among adults in the United States, 2014–2018. JAMA. 2019; 322(18):1824–1827. DOI: 10.1001/jama.2019.15331 31524940 PMC6749536

[65] Skotsimara G, Antonopoulos AS, Oikonomou E, Siasos G, Ioakeimidis N, Tsalamandris S, et al. Cardiovascular effects of electronic cigarettes: A systematic review and meta-analysis. European Journal of Preventative Cardiology. 2019; 26(11):1219–1228. DOI: 10.1177/2047487319832975 30823865

[66] Wang JB, Olgin JE, Nah G, Vittinghoff E, Cataldo JK, Pletcher MJ, et al. Cigarette and e-cigarette dual use and risk of cardiopulmonary symptoms in the Health eHeart Study. PLoS One. 2018; 13(7):e0198681e0198681. DOI: 10.1371/journal.pone.0198681 30044773 PMC6059385

[67] Ip M, Diamantakos E, Haptonstall K, Choroomi Y, Moheimani RS, Nguyen KH, et al. Tobacco and electronic cigarettes adversely impact ECG indexes of ventricular repolarization: Implication for sudden death risk. American Journal of Physiology Heart and Circulatory Physiology. 2020; 318(5):H1176–H1184. DOI: 10.1152/ajpheart.00738.2019 32196360 PMC7346537

[68] Caporale A, Langham MC, Guo W, Johncola A, Chatterjee S, Wehrli FW. Acute effects of electronic cigarette aerosol inhalation on vascular function detected at quantitative MRI. Radiology. 2019; 293(1):97–106. DOI: 10.1148/radiol.2019190562 31429679 PMC6776371

[69] Fetterman JL, Keith RJ, Palmisano JN, McGlasson KL, Weisbrod RM, Majid S, Bastin R, et al. Alterations in vascular function associated with the use of combustible and electronic cigarettes. Journal of the American Heart Association. 2020; 9(9):e014570. DOI: 10.1161/JAHA.119.014570 32345096 PMC7428567

[70] Klaas FJW, Silja CT, Moritz M, Friedhelm S, Michael R, Klaus D, et al. E-cigarettes and cigarettes worsen peripheral and central hemodynamics as well as arterial stiffness: a randomized, double-blinded pilot study. Vascular Medicine. 2018; 23(5):419–425. DOI: 10.1177/1358863X18779694 29985113

[71] Espinoza-Derout J, Shao XM, Lao CJ, Hasan KM, Rivera JC, Jordan MC, et al. Electronic cigarette use and the risk of cardiovascular diseases. Frontiers in Cardiovascular Medicine. 2022; 9:879726. DOI: 10.3389/fcvm.2022.879726 35463745 PMC9021536

[72] Kim SY, Sim S, Choi HG. Active, passive, and electronic cigarette smoking is associated with asthma in adolescents. Scientific Reports. 2017; 7(1):17789. DOI: 10.1038/s41598-017-17958-y 29259221 PMC5736689

[73] de Borba AT, Jost RT, Gass R, Nedel FB, Cardoso DM, Pohl HH, et al. The influence of active and passive smoking on the cardiorespiratory fitness of adults. Multidisciplinary Respiratory Medicine. 2014; 9(1):34. DOI: 10.1186/2049-6958-9-34 25009739 PMC4088222

[74] Hom S, Chen L, Wang T, Ghebrehiwet B, Yin W, Rubenstein DA. Platelet activation, adhesion, inflammation, and aggregation potential are altered in the presence of electronic cigarette extracts of variable nicotine concentrations. Platelets. 2016; 27(7):694–702. DOI: 10.3109/09537104.2016.1158403 27096416

[75] Rader F, Rashid M, Nguyen TT, Luong E, Kim A, Kim E, et al. E-cigarette use and subclinical cardiac effects. Circulation Research. 2020; 127(12):1566–1567. DOI: 10.1161/CIRCRESAHA.120.316683 33043813 PMC8049525

[76] Sifat AE, Vaidya B, Kaisar MA, Cucullo L, Abbruscato TJ. Nicotine and electronic cigarette (e-cig) exposure decreases brain glucose utilization in ischemic stroke. Journal of Neurochemistry. 2018; 147(2):204–221. DOI: 10.1111/jnc.14561 30062776 PMC6394831

[77] Raval AP. Nicotine addiction causes unique detrimental effects on women’s brains. Journal of Addictive Diseases. 2011; 30(2):149–158. DOI: 10.1080/10550887.2011.554782 21491296

[78] Moheimani RS, Bhetraratana M, Yin F, Peters KM, Gornbein J, Araujo JA, et al. Increased cardiac sympathetic activity and oxidative stress in habitual electronic cigarette users: Implications for cardiovascular risk. JAMA Cardiology. 2017; 2(3):278–284. DOI: 10.1001/jamacardio.2016.5303 28146259 PMC5626008

[79] Basch CH, Mongiovi J, Hillyer GC, MacDonald Z, Basch CE. YouTube™ videos related to e-cigarette safety and related health risks: Implications for preventing and emerging epidemic. Public Health. 2016; 132:57–59. DOI: 10.1016/j.puhe.2015.12.003 26826891

[80] Laranjo L, Lanas F, Sun MC, Chen DA, Hynes L, Imran TF, et al. World Heart Federation roadmap for secondary prevention of cardiovascular disease: 2023 update. Global Heart. 2024; 19(1):8. DOI: 10.5334/gh.1278 38273995 PMC10809857

[81] Fiore MC, Jaén CR, Baker TB, Bailey WC, Benowitz NL, Curry SJ, et al. Treating tobacco use and dependence: 2008 update. Rockville, MD: US Department of Health and Human Services; 2008 May. 276 p.

[82] Rakel RE, Houston T. Nicotine addiction. In Rakel RE, Rakel DP (Eds.) Textbook of Family Medicine. Philadelphia, PA: Elsevier Health Sciences; 2011. pp. 1105–1122. DOI: 10.1016/B978-1-4377-1160-8.10050-8

[83] Twyman L, Bonevski B, Paul C, Bryant J. Perceived barriers to smoking cessation in selected vulnerable groups: A systematic review of the qualitative and quantitative literature. BMJ Open. 2014; 4(12):e006414. DOI: 10.1136/bmjopen-2014-006414 PMC4275698 25534212

[84] Smokefree.gov. Cessation services data [Internet]. National Cancer Institute; [cited 2024 Dec 14]. Available from: https://smokefree.gov/about-us/cessation-services-data#:~:text=National%20Cessation%20Access%20Portals&text=The%20NNTCQ%20connects%20persons%20interested,%2D855%2DDEJELO%2DYA.

[85] Centers for Disease Control and Prevention. Quitlines and other cessation support resources [Internet]. Centers for Disease Control and Prevention; [updated 2024 May 15; cited 2024 Dec 15]. Available from: https://www.cdc.gov/tobacco/hcp/patient-care/quitlines-and-other-resources.html.

[86] National Health Service. NHS stop smoking services help you quit [Internet]. National Health Service; [updated 2022 Aug 17; cited 2024 Dec 15]. Available from: https://www.nhs.uk/live-well/quit-smoking/nhs-stop-smoking-services-help-you-quit/.

[87] Nides M. Update on pharmacologic options for smoking cessation treatment. The American Journal of Medicine. 2008; 121(4):S20–S31. DOI: 10.1016/j.amjmed.2008.01.016 18342163

[88] Hays JT, McFadden DD, Ebbert JO. Pharmacologic agents for tobacco dependence treatment: 2011 update. Current Atherosclerosis Reports. 2012; 14(1):85–92. DOI: 10.1007/s11883-011-0211-2 22002681

[89] Eisenberg MJ, Filion KB, Yavin D, Belisle P, Mottillo S, Joseph L, et al. Pharmacotherapies for smoking cessation: A meta-analysis of randomized controlled trials. CMAJ. 2008; 179(2):135–144. DOI: 10.1503/cmaj.070256 18625984 PMC2443223

[90] Stead L, Perera R, Bullen C, Mant D, Cahill K, Lancaster T. Nicotine replacement therapy for smoking cessation. The Cochrane Database of Systematic Reviews. 2012; 11:CD000146. DOI: 10.1002/14651858.CD000146.pub4 18253970

[91] Hughes J, Stead L, Cahill K, Lancaster T. Antidepressants for smoking cessation. The Cochrane Database of Systematic Reviews. 2007; 1:CD00031. DOI: 10.1002/14651858.CD000031.pub3 17253443

[92] Center for Drug Evaluation and Research. FDA Drug Safety Communication: FDA revises description of Mental Health [Internet]. U.S. Food and Drug Administration; 2018 [cited 2025 Feb 15]. Available from: https://www.fda.gov/drugs/drug-safety-and-availability/fda-drug-safety-communication-fda-revises-description-mental-health-side-effects-stop-smoking.

[93] Evins AE, Benowitz NL, West R, Russ C, McRae T, Lawrence D, et al. Neuropsychiatric safety and efficacy of varenicline, bupropion, and nicotine patch in smokers with psychotic, anxiety, and mood disorders in the EAGLES trial. Journal of Clinical Psychopharmacology. 2019; 39(2):108–116. DOI: 10.1097/JCP.0000000000001015 30811371 PMC6488024

[94] Cahill K, Lindson-Hawley N, Thomas K, Fanshawe T, Lancaster T. Nicotine receptor partial agonists for smoking cessation. The Cochrane Database Systematic Reviews. 2016; 5:CD006103. DOI: 10.1002/14651858.CD006103.pub7 PMC6464943 27158893

[95] Center for Drug Evaluation and Research. Safety Review update of Chantix [Internet]. U.S. Food and Drug Administration; 2017 [cited 2025 Feb 15]. Available from: https://www.fda.gov/drugs/drug-safety-and-availability/fda-drug-safety-communication-safety-review-update-chantix-varenicline-and-risk-cardiovascular.

[96] US Food and Drug Administration. FDA drug safety communication: Chantix (varenicline) may increase the risk of certain cardiovascular adverse events in patients with cardiovascular disease [Internet]. US Food and Drug Administration; [updated 2016 Mar 2; cited 2017 Jul 5]. Available from: https://www.fda.gov/drugs/drug-safety-and-availability/fda-drug-safety-communication-chantix-varenicline-may-increase-risk-certain-cardiovascular-adverse.

[97] Dehghani P, Habib B, Windle SB, Roy N, Old W, Grondin FR, et al. Smokers and postcessation weight gain after acute coronary syndrome. Journal of the American Heart Association. 2017; 6(4):e004785. DOI: 10.1161/JAHA.116.004785 PMC5532997 28420644

[98] Gourlay S, Stead L, Benowitz N. Clonidine for smoking cessation. The Cochrane Database of Systematic Reviews. 2004; 3:CD000058. DOI: 10.1002/14651858.CD000058.pub2 PMC7038651 15266422

[99] Hughes JR, Stead LF, Lancaster T. Nortriptyline for smoking cessation: A review. Nicotine & Tobacco Research. 2005; 7(4):491–499. DOI: 10.1080/14622200500185298 16085520

[100] Tinghino B, Cardellicchio S, Corso F, Cresci C, Pittelli V, Principe R, et al. Cytisine for smoking cessation: A 40-day treatment with an induction period. Tobacco Prevention & Cessation. 2024; 10. DOI: 10.18332/tpc/187556 PMC11129281 38803387

[101] Blanch P, Freixa-Pamais R. When are you going to quit smoking? European Society of Cardiology. 2021; 20(6). Available from: https://www.escardio.org/Journals/E-Journal-of-Cardiology-Practice/Volume-20/when-are-you-going-to-quit-smoking.

[102] European Network for Smoking and Tobacco Prevention. European smoking cessation guidelines and quality standards [Internet]. European Network for Smoking and Tobacco Prevention; [cited 2024 Sept 11]. Available from: https://ensp.network/european-smoking-cessation-guidelines-and-quality-standards/.

[103] Rigotti NA, Benowitz NL, Prochaska J, Leischow S, Nides M, Blumenstein B, et al. Cytisinicline for smoking cessation: A randomized clinical trial. JAMA. 2023; 330(2):152–160. DOI: 10.1001/jama.2023.10042 37432430 PMC10336611

[104] Lindson N, Chepkin SC, Ye W, Fanshawe TR, Bullen C, Hartmann-Boyce J, et al. Different doses, durations and modes of delivery of nicotine replacement therapy for smoking cessation. The Cochrane Database of Systematic Reviews. 2019; 4:CD013308. DOI: 10.1002/14651858.CD013308 30997928 PMC6470854

[105] Hajizadeh A, Howes S, Theodoulou A, Klemperer E, Hartmann-Boyce J, Livingstone-Banks J, et al. Antidepressants for smoking cessation. The Cochrane Database of Systematic Reviews. 2023; 5(5):CD000031. DOI: 10.1002/14651858.CD000031.pub6 37230961 PMC10207863

[106] Cinciripini PM, Green CE, Shete S, Minnix JA, Robinson JD, Cui Y, et al. Smoking cessation after initial treatment failure with varenicline or nicotine replacement: A randomized clinical trial. JAMA. 2024; 331(20):1722–1731. DOI: 10.1001/jama.2024.4183 38696203 PMC11066767

[107] Fiore MC. Tobacco use and dependence: A 2011 update of treatments [Internet]. University of Wisconsin School of Medicine and Public Health [updated 2012 Jan 25]. Available from: https://www.medscape.org/viewarticle/757167.

[108] Ockene I, Salmoirago-Blotcher E. Varenicline for smoking cessation in patients with coronary heart disease. Circulation. 2010; 121(2):188–90. DOI: 10.1161/CIRCULATIONAHA.109.915546 20048200

[109] Dinno A, Glantz S. Clean indoor air laws immediately reduce heart attacks. Preventive Medicine. 2007; 45(1):9–11. DOI: 10.1016/j.ypmed.2007.03.013 17499350 PMC2693058

[110] Barua R, Rigotti N, Benowitz N, Cummings KM, Jazayeri MA, Morris PB, et al. ACC expert consensus decision pathway on tobacco cessation treatment: A report of the American College of Cardiology Task Force on Clinical Expert Consensus Documents. Journal of the American College of Cardiology. 2018; 72(25):3332–3365. DOI: 10.1016/j.jacc.2018.10.027 30527452

[111] Müezzinler A, Mons U, Gellert C, Schöttker B, Jansen E, Kee F, et al. Smoking and all-cause mortality in older adults: Results from the CHANCES consortium. American Journal of Preventative Medicine. 2015; 49(5):e53–e63. DOI: 10.1016/j.amepre.2015.04.004 26188685

[112] Bolliger CT, Zellweger JP, Danielsson T, van Biljon X, Robidou A, Westin A, et al. Influence of long-term smoking reduction on health risk markers and quality of life. Nicotine & Tobacco Research. 2002; 4(4):433–439. DOI: 10.1080/1462220021000018380.96 12521402

[113] Aubin HJ, Farley A, Lycett D, Lahmek P, Aveyard P. Weight gain in smokers after quitting cigarettes: Meta-analysis. BMJ. 2012; 345:e4439. DOI: 10.1136/bmj.e4439 22782848 PMC3393785

[114] Wada H, Ura S, Satoh-Asahara N, Kitaoka S, Mashiba S, Akao M, et al. α1-antitrypsin low-density-lipoprotein serves as a marker of smoking-specific oxidative stress. Journal of Atherosclerosis and Thrombosis. 2012(1);19:47–58. DOI: 10.5551/jat.9035 22027559

[115] Komiyama M, Wada H, Ura S, Yamakage H, Satoh-Asahara N, Shimada S, et al. The effects of weight gain after smoking cessation on atherogenic α1-antitrypsin low-density lipoprotein. Heart and Vessels. 2015; 30(6):734–739. DOI: 10.1007/s00380-014-0549-9 25086816 PMC4648963

[116] Komiyama M, Shimada S, Wada H, Yamakage H, Satoh-Asahara N, Shimatsu A, et al. Time-dependent changes of atherosclerotic LDL complexes after smoking cessation. Journal of Atherosclerosis and Thrombosis. 2016; 23(11):1270–1275. DOI: 10.5551/jat.34280 27298048 PMC5113744

[117] Hu Y, Zong G, Liu G, Wang M, Rosner B, Pan A, et al. Smoking cessation, weight change, type 2 diabetes, and mortality. The New England Journal of Medicine. 2018; 379:623–32. DOI: 10.1056/NEJMoa1803626 30110591 PMC6165582

[118] Bush T, Lovejoy J, Javitz H, Torres AJ, Wassum K, Tan MM, et al. Simultaneous vs. sequential treatment for smoking and weight management in tobacco quitlines: 6 and 12 month outcomes from a randomized trial. BMC Public Health. 2018; 18:678–690. DOI: 10.1186/s12889-018-5574-7 29855294 PMC5984316

